# Gut Microbes and Hepatic Encephalopathy: From the Old Concepts to New Perspectives

**DOI:** 10.3389/fcell.2021.748253

**Published:** 2021-11-26

**Authors:** Alba Rocco, Costantino Sgamato, Debora Compare, Pietro Coccoli, Olga Maria Nardone, Gerardo Nardone

**Affiliations:** Gastroenterology and Hepatology, Department of Clinical Medicine and Surgery, University Federico II, Naples, Italy

**Keywords:** hepatic encephalopathy, gut microbes, gut-liver-brain axis, antibiotics, probiotics, fecal microbiota transplantation

## Abstract

Hepatic encephalopathy (HE) is a severe complication of advanced liver disease and acute liver failure. The clinical spectrum ranges from minor cognitive dysfunctions to lethargy, depressed consciousness, and coma and significantly impact the quality of life, morbidity, and mortality of the patients. It is commonly accepted that the gut milieu is essential for the development of HE; however, despite intensive research efforts, the pathogenesis of HE is still not fully elucidated. As our knowledge of gut microbiota moves from the pioneering era of culture-dependent studies, the connection between microbes, inflammation, and metabolic pathways in the pathogenesis of HE is becoming increasingly clear, providing exciting therapeutic perspectives. This review will critically examine the latest research findings on the role of gut microbes in the pathophysiological pathways underlying HE. Moreover, currently available therapeutic options and novel treatment strategies are discussed.

## Introduction

Hepatic encephalopathy (HE) encompasses a broad spectrum of neurological or psychiatric abnormalities ranging from minor cognitive dysfunction to lethargy, depressed consciousness, and coma occurring in patients with liver insufficiency or portosystemic shunting (Vilstrup et al., 2014). According to the severity of the clinical presentation, HE has traditionally been classified into overt (OHE) and minimal HE (MHE). Clinically manifest neurological-psychiatric abnormalities characterise OHE, while neuropsychological or electrophysiological alterations without clinically detectable abnormalities are typical of MHE (Ferenci et al., 2002). HE is likely the most frequent complication of cirrhosis, with a prevalence ranging from 16 to 21% for OHE in decompensated cirrhosis to 20–80% for MHE in compensated cirrhosis (D’Amico et al., 1986; Allampati et al., 2016). The onset of HE is associated with a high risk of recurrence, hospital admission rate, and poor survival and impacts the quality of life of patients and their caregivers (Cordoba et al., 2014).

Despite intensive research efforts, the pathogenesis of HE is still not fully elucidated. Thus, effective therapies for treating and preventing HE are still lacking, hence the urgent need to update our knowledge, moving from the old concepts to the newest perspectives.

### From the Ammonia Hypothesis to the Gut-Liver-Brain Axis

It is commonly accepted that neurological impairment and cognitive decline provoked by liver dysfunction result from blood-derived factors influencing the permeability and altering the integrity of the blood-brain barrier. Since the description of the “meat intoxication syndrome” in portocaval-shunted dogs, at the end of the 19th century, ammonia has been considered the critical metabolic factor underlying HE’s development (Amodio, 2015).

Ammonia primarily derives from the gut as an end product of protein digestion, amino acid deamination, and bacterial urease activity. Furthermore, multiple organs, such as the brain, muscle, and kidney, contribute to ammonia production by deaminating glutamine via glutaminase. In physiological condition, the liver efficiently extracts (85%) the ammonia from portal blood and, through the urea cycle, convert it into urea, then excreted in the kidneys (75%) and the intestine (25%). Only 15% of the ammonia pool enters the systemic circulation (Rose et al., 2020). Defects in hepatic function, portal blood flow, and urea cycle enzymes or intermediates can result in hyperammonemia, as can excessive ammonia production in the gastrointestinal tract. The ammonia passes freely into the brain, where astrocytes remove it producing glutamine via glutamine synthetase. Glutamine induces astrocyte hypertonia resulting in astrocyte swelling, compromised neuronal communication, impaired function, and brain edema. However, ammonia concentration and HE severity are poorly correlated, thus demonstrating that it is only a piece of the puzzle underpinning the pathogenesis of HE (Shawcross et al., 2011). In addition to the direct role of ammonia, systemic inflammation/oxidative stress and increased blood bile acids impact the blood-brain barrier permeability, allowing an increased influx of molecules physiologically unable to cross it (Atluri et al., 2011). Consequently, the alterations of metabolites in cerebrospinal fluid and changes in neurotransmission such as increased GABAergic tone significantly modulate the onset of neurological decline (Keiding et al., 2006; Riordan and Williams, 2010). More recently, neurosteroids, endogenous benzodiazepines, and manganese have emerged as synergistic factors in the onset of HE (Rose et al., 2020). Furthermore, like a vicious circle, hyperammonaemia “*per se*” can induce neutrophil dysfunction and reactive oxygen species release, contributing to systemic oxidative stress and inflammation, exacerbating its harmful effects in the brain (Shawcross et al., 2004).

The gut-liver-brain axis refers to the bidirectional relationship between the gut and its microbiota, the liver, and the brain, resulting from integrating signals generated by dietary, genetic, and environmental factors (Mancini et al., 2018). In patients with liver cirrhosis, defective small intestinal motility, reduced gastric acid secretion, and weaker antimicrobial defense of intestinal mucosa determine small intestinal bacterial overgrowth (SIBO). The concomitant decrease in bile acids (BAs) synthesis due to liver failure synergistically act with SIBO to determine pathological changes in the intestinal microbiota composition, mainly characterised by a massive reduction in microbial diversity, a decline in autochthonous non-pathogenic bacteria (*Bacteroidetes*, *Ruminococcus*, *Roseburia*, *Veillonellaceae*, and *Lachnospiraceae*) and an overgrowth of potentially pathogenic species (*Fusobacteria*, *Proteobacteria*, *Enterococcaceae*, and *Streptococcaceae*) (Fukui, 2015; Acharya and Bajaj, 2017).

The paucity of bacteria involved in producing short-chain fatty acids (SCFAs) and converting primary into secondary BAs contribute to worsening gut dysbiosis and disrupting intestinal barrier integrity. Indeed, SCFAs (mainly butyrate, acetate, and propionate), produced in the colon by bacterial fermentation of dietary fibers and resistant starch, exert several beneficial functions: preserving intestinal barrier integrity, nourishing colonocytes promoting, mucus production, and reducing of colonic inflammation (Nava and Stappenbeck, 2011; Rowland et al., 2018). Furthermore, the lower abundance of 7α-dehydroxylating bacteria in the colon (*Lachonospiraceae*, *Ruminococcaceae*, and *Blautia*) due to a reduction in primary BAs determines a change in secondary-to-primary BAs ratio that can favor the overgrowth of pathogenic taxa (Kakiyama et al., 2013; Ridlon et al., 2013).

The impaired intestinal barrier integrity enhances bacterial translocation and the release of bacterial endotoxins in circulation, such as lipopolysaccharides, flagellin, peptidoglycan, and microbial nucleic acids, perpetuating liver damage and contributing to systemic inflammation responsible for blood-brain barrier dysfunction and neuroinflammation (Shawcross et al., 2004; Shawcross et al., 2007; Shawcross et al., 2011; Dhiman, 2012).

Dysregulation of blood-brain barrier permeability may also lead to a dramatic increase of certain BAs such as lithocholic, taurocholic, and glycocholic acid in the cerebrospinal fluid of patients with HE or brain tissue of rodent model of HE (Tripodi et al., 2012; McMillin et al., 2016; Weiss et al., 2016).

Although a precise role for BAs in the pathogenesis of HE has not yet been completely defined, they are likely involved in aberrant neuronal signalling and the promotion of neuroinflammation through microglia activation (DeMorrow, 2019).

**FIGURE 1:**
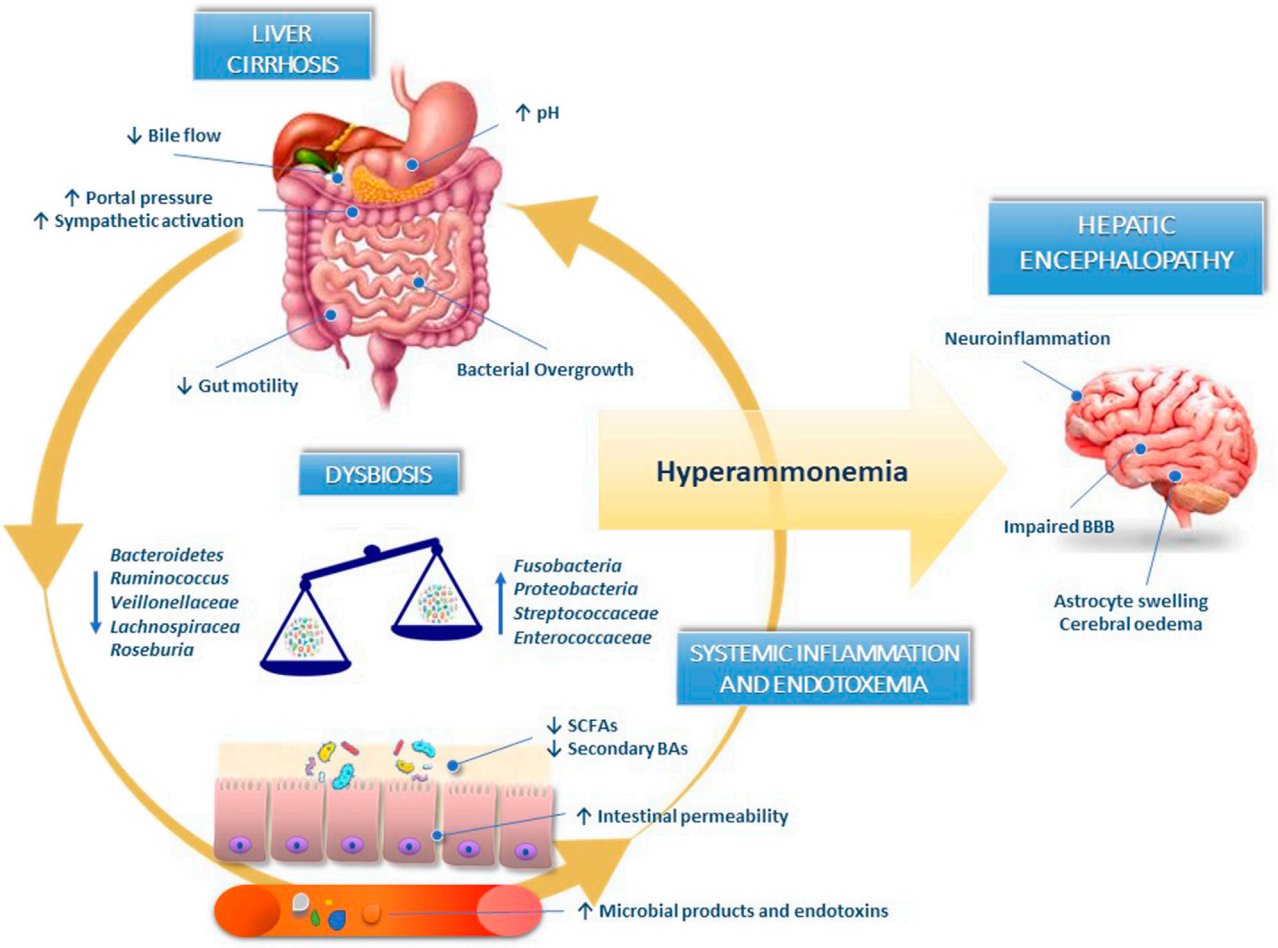
Gut-liver-brain axis in the pathogenesis of hepatic encephalopathy. In liver cirrhosis, the decrease in bile acids synthesis, defective small intestinal motility and reduced gastric acid secretion induce small intestinal bacterial overgrowth and dysbiosis. The reduced abundance of bacteria synthesising short-chain fatty acids and converting primary into secondary bile acids contribute to worsening gut dysbiosis and disrupting intestinal barrier integrity. Pathological bacterial translocation and release of bacterial endotoxins in circulation perpetuate liver damage and contribute to systemic inflammation responsible for blood-brain barrier dysfunction and neuroinflammation. BAs, bile acids; BBB, blood-brain barrier; SCFAs, short-chain fatty acids.

Overall, HE can be considered a typical gut-liver-brain axis disease model (Figure 1).

## Gut Microbiota in Liver Cirrhosis

### Culture-Based Studies on Gut Microbiome in Human Cirrhosis

First attempts to characterise gut microbiota composition employed culture-based technologies and analysed microbial changes after HE therapy (Table 1). Riggio et al. observed a significant growth, defined as more than 2 log increases of non-urease producing *Lactobacilli* spp. after both lactulose and lactitol treatment and a reduction in proteolytic bacteria (*Enterobacteria* and *Enterococci*) after lactitol alone (Riggio et al., 1990). Lactitol administration for 4 weeks was also associated with an increased occupation ratio (number of specific bacteria/total number of bacteria detected) of anaerobic *Bifidobacterium* and a rise in *Lactobacilli* total count. Furthermore, a reduction in *Clostridium* and *Bacteroides*, considered to be ammonia-producing bacteria, was observed. These changes in gut microbiota paralleled the decrease in venous ammonia level and improvement of mental status and asterixis (Tarao et al., 1995). Last, in a randomised, placebo-controlled trial involving 55 patients with MHE, Liu et al. demonstrated that a symbiotic treatment with probiotics and fermentable fiber effectively increased the fecal content of *Lactobacillus* spp. at the expense of the overgrowth of pathogenic bacteria, such as *Escherichia coli* and *Staphylococcus* spp. Symbiotic treatment was further associated with reduction of serum ammonia and reversal of MHE in 50% of the patients compared to the placebo group (Liu et al., 2004). Although the data derived from these pivotal studies were the cornerstones of the “microbial revolution” in the pathogenesis of chronic liver diseases and HE, culture-based methodologies used to characterise the microbial communities hamper the results. Indeed, most bacterial species inhabiting the gut can either not be cultured or reliably distinguished. Further, these techniques are only qualitative or, at best, semi-quantitative.

**TABLE 1:** Culture-based studies on gut microbiome in human cirrhosis.

Author	Population	Sample	Methods	Results
Riggio et al. (1990)	Cirrhotic patients	Stool	Culture	↑ *Lactobacilli* spp. after both lactulose and lactitol therapy
				↓ *Enterobacteria* and *Enterococci* after lactitol
Tarao et al. (1995)	Cirrhosis with HE	Stool	Culture	↑ Occupation ratio of *Bifidobacterium* and ↓ *Clostridium* and *Bacteroides* after lactitol treatment
				↓ Serum ammonia and improvement of mental status and asterixis
Liu et al. (2004)	Cirrhosis with HE	Stool	Culture	↑ *Lactobacillus* spp. and ↓ Escherichia *coli* and S*taphylococcus* spp.
				↓ Serum ammonia and reversal of MHE in 50% of patients

### Culture-Independent Studies on Gut Microbiome in Human Cirrhosis

The introduction of culture-independent techniques has revolutionised the field of intestinal microbiology (Table 2). Through the sequencing of the bacterial 16S ribosomal RNA (16S rRNA) gene containing variable regions useful for phylogenetic identification, more recent studies better defined the taxonomic profile of gut microbiota in patients affected by chronic liver diseases in respect to healthy individuals (Fraher et al., 2012; Qin et al., 2014).

**TABLE 2:** Culture-independent studies on gut microbiome in human cirrhosis with or without HE.

Author	Population	Sample	Methods	Results
Chen et al. (2011)	Cirrhosis vs healthy control	Stool	16S sequencing	*Proteobacteria* and *Fusobacteria* phyla and *Streptococcaceae*, *Veillonellaceae* and *Enterobacteriaceae* families higher in cirrhotic patients than in controls
				*Bacteroidetes* phylum and *Lachnospiraceae* family lower in cirrhotic patients than in controls
Bajaj et al. (2014b)	Cirrhosis vs healthy controls	Stool	16S sequencing, MTPS	↓ Autochthonous taxa (*Lachnospiraceae*, *Ruminococcaceae*, and Clostridiales XIV), non-autochthonous taxa (*Enterobacteriaceae* and *Bacteroideaceae*) ratio
Bajaj et al. (2012b)	OHE/non-OHE/control	Stool	16S sequencing, MTPS	*↑Enterobacteriaceae*, *Alcaligeneceae*, and *Fusobacteriaceae* and *↓ Ruminococcaceae* and *Lachnospiraceae* in cirrhotic group versus controls
				↑ *Enterobacteriaceae*, *Alcaligenaceae*, *Lactobacilaceae*, and *Streptococcaceae* in OHE versus controls
				↑ *Veillonellaceae* in OHE versus no OHE
				*Alcaligeneceae* and *Porphyromonadaceae* associated with poor cognition
Bajaj et al. (2012a)	OHE/no-OHE/control	Stool Sigmoid mucosa	16S sequencing	↑*Dorea*, *Subdoligranulum*, Incertae Sedis XIV, *Blautia*, *Roseburia*, *Faecalibacterium* and ↓ *Enterococcus*, *Burkholderia*, *Proteus* in cirrhosis
				↑ *Enterococcus*, *Veillonella*, *Megasphaera*, and *Burkholderia* and ↓ *Roseburia* in OHE mucosal microbiome
Zhang et al. (2013)	MHE/no MHE/control	Stool	16S sequencing	*↑ Veillonellaceae* and *Streptococcaceae* in cirrhotics
				*Streptococcus salivarius* was higher in MHE
				*Veillonella parvula* and *Streptococcus salivarius* were correlated with cognitive function and ammonia level
Bajaj et al. (2015a)	Previous HE/non-HE/control	Saliva	16S sequencing	↑*Enterobacteriaceae*, *Enterococcacea* and ↓ in autochthonous microbiota and *Erysipelothricaceae* in previous HE, compared to non-HE patients and controls
Ahluwalia et al. (2016)	Previous HE/non-HE/control	Stool	16S sequencing	*Streptococcaceae*, *Enterobacteriaceae*, *Lactobacillaceae* and *Peptostreptococcacea*e, were positively linked with hyperammonemia-associated astrocytic changes
				*Porphyromonadaceae*, were correlated with neuronal integrity and oedema
Iebba et al. (2018)	Cirrhosis vs control	Stool	16S sequencing and NMR metabolism	*Stenotrophomonas pavanii*, *Methylobacterium* as well as metabolites (methanol, threonine), enhanced the risk of HE
Sung et al. (2019)	AHE vs. cirrhosis/control	Stool	16S sequencing	*↑ Firmicute*, *Proteobacteria* and *Actinobacteria* during AHE
				*Alistipes*, *Bacteroides*, *Phascolarctobacterium* were associated with HE recurrence
				*Clostridium-XI*, *Bacteroides*, *Bacteroides*, *Lactobacillus*, *Clostridium sedis* were associated with overall survival at 1-year follow-up
Bloom et al. (2021)	Previous OHE/no-OHE	Stool	Shotgun sequencing and LC-MS/MS	*Anaeromassilibacillus species*, *Anaerostipes caccae*, *Bacteroideseggerthii*, *Clostridium species*, *Faecalicatena contorta*, *Holdemaniafiliformis*, *Neglecta timonensis*, and *Ruminococcus* species were linked to a history of OHE
				Lower concentrations of 6 faecal SCFAs in patients with a history of OHE

OHE, overt hepatic encephalopathy; MHE, minimal hepatic encephalopathy; AHE, acute hepatic encephalopathy; NMR, nuclear magnetic resonance; LC-MS/MS, liquid chromatography tandem mass spectrometry.

Chen et al. first characterised fecal microbial communities in patients with liver cirrhosis using pyrosequencing of the 16S rRNA V3 region (Chen et al., 2011). Compared to healthy individuals, cirrhotic patients had lower microbial diversity, as estimated by the Shannon diversity index, and changes in the intestinal microbial community composition both in terms of phyla (with a marked decrease in the relative abundance of *Bacteroidetes* and enrichment in *Proteobacteria* and *Fusobacteria*) and families (enrichment in *Enterobacteriaceae*, *Pasteurellaceae*, *Streptococcaceae*, *Veillonellaceae* and depletion in *Lachnospiraceae*). Interestingly, *Streptococcaceae* showed a positive correlation trend, whereas *Lachnospiraceae* negatively correlated with the severity of cirrhosis assessed by the Child-Pugh score. The enrichment of *Streptococcus*, *Veillonella*, and *Enterobacteriaceae* in fecal microbiota might result from a relocation of small intestinal bacteria. On the other hand, a decline in species involved in SCFAs metabolism, such as *Lachnospiraceae*, could lead to a higher colonic pH and ammonia production (Justesen et al., 1984; Vince et al., 1990).

In a more extensive study involving 244 patients covering the spectrum from healthy controls to decompensated cirrhosis, the authors found a progressive decrease in the ratio between potentially beneficial autochthonous taxa (*Lachnospiraceae*, *Ruminococcaceae*, and Clostridiales XIV) and harmful non-autochthonous taxa (*Enterobacteriaceae* and *Bacteroideaceae*), so-called cirrhosis dysbiosis ratio, paralleling the worsening of liver disease and higher endotoxemia (Bajaj et al., 2014). Thus, the imbalance of intestinal microbiota composition negatively affects the natural history of liver disease leading to hepatic and extra-hepatic complications.

### Gut Microbiota and Hepatic Encephalopathy

Compared with cirrhotic patients without cognitive dysfunction, patients with both MHE and OHE had specific alterations of gut microbiota profile. Bajaj et al. firstly demonstrated that the differences in stool microbiome composition between healthy controls and cirrhotic were more pronounced analysing the results according to HE. In detail, the abundance of *Lachnospiraceae* and *Ruminococceae* was significantly higher in the control group, whereas *Enterobacteriaceae*, *Fusobacteriaceae*, *Alcaligenaceae*, *Lactobacillaceae*, and *Leuconostocaceae* were significantly lower in the controls compared with cirrhotic patients, irrespective of HE. However, the HE group differed from controls on several additional bacterial families compared with cirrhotics without HE with a significantly higher concentration of *Enterobacteriaceae*, *Alcaligenaceae*, *Lactobacilaceae*, and *Streptococcaceae*. Moreover, altered flora (higher *Veillonellaceae*), poor cognition, endotoxemia, and inflammation (IL-6, TNF-α, IL-2, and IL-13) were observed in HE compared with cirrhotics without HE (Bajaj et al., 2012b). More strikingly, the authors found that specific bacterial families (*Alcaligeneceae*, *Porphyromonadaceae*, *Enterobacteriaceae*) were strongly associated with both cognition and inflammation in HE. *Alcaligeneceae*, in particular, can produce ammonia by degradation of urea, likely explaining the correlation with cognitive impairment (Bajaj et al., 2012b). Later, the same authors analysed both the stool and colonic mucosal microbiome of 60 cirrhotic patients. The sigmoid mucosal microbiome considerably differed from the corresponding stool samples, and these differences persisted in studying the group according to the presence of HE. In detail, members of genera *Enterococcus*, *Megasphaera*, and *Burkholderia* were overrepresented in HE and linked to poor cognition and inflammation, whereas *Roseburia* prevailed in the group without HE. The alteration of bile acid metabolism and the decrease of antibacterial peptides or mucins in the colon, typically occurring in the advanced stages of liver diseases, could lead to a selection of potentially pathogenic bacteria adhering to and growing in the colonic mucosa. Thus, several essential processes in the pathogenesis of HE probably occur at the mucosal surface rather than lumens, such as translocation and interaction between microbiota and the immune system (Bajaj et al., 2012a).

Zhang et al. found that the stool concentration of the gut urease-containing bacteria *Streptococcus salivarius* was significantly higher in cirrhotic patients with MHE than in those without HE. Furthermore, the change in the amount of these bacteria positively correlated with ammonia accumulation (Zhang et al., 2013). The difference in the bacterial families associated with HE reported by the authors can be explained by the high interindividual variations in gut microbiota across populations, or other unknown factors could (Yatsunenko et al., 2012).

Interestingly, dysbiosis, resulting from decreased autochthonous or commensal taxa, has also been found in the saliva of patients with cirrhosis compared to controls. Qin et al. reported that most of the enteral consortia detectable in cirrhotic (mainly *Streptococcus* spp. and *Veillonella* spp.) belong to the oropharyngeal inhabitants, suggesting an invasion of the gut by oral bacterial species (Qin et al., 2014). Salivary microbiome analysis showed an increase in pathogenic *Enterobacteriaceae* and a reduction in autochthonous microbiota *Erysipelothricaceae* in HE, compared to non-HE patients and controls, thus indicating a global mucosal-immune interface alteration (Bajaj et al., 2015a).

Gut dysbiosis can also directly impact brain homeostasis, with neuronal and astrocytic dysfunction, particularly in HE. Ahluwalia et al., using multi-modal magnetic resonance imaging (MRI), correlated specific microbial families with neuroradiological findings. Hyperammonemia-associated astrocytic changes (i.e., increased glutamate/glutamine ratio and reduced myo-inositol and choline) at the magnetic resonance spectroscopy (MRS) positively correlated with families *Streptococcaceae*, *Enterobacteriaceae*, *Lactobacillaceae*, and *Peptostreptococcaceae*, while negatively correlated with *Lachospiraceae*, *Ruminococcaeae*, and Clostridiales XIV. On the other hand, *Porphyromonadaceae* were only associated with neuronal changes on diffusion tensor imaging, used to assess neuronal integrity and edema, without linkages with ammonia (Ahluwalia et al., 2016).

Despite the impressive results, a more comprehensive microbiota analysis should combine metagenomics with other “omics” approaches, particularly metatranscriptomics and metabolomics. Metatranscriptomics allows understanding gene expression and protein activity, whereas metabolomics represents the comprehensive analysis of metabolites released of the entire micro¬bial community. Iebba et al. made one of the first attempts to integrate these different approaches. Through the combination of 16s DNA sequencing, nuclear magnetic resonance (NMR) metabolomics, and network analysis, they observed that the translocation of certain species (*Stenotrophomonas pavanii*, *Methylobacterium extorquens*) into the peripheral blood system, as well as metabolites (methanol, threonine), enhanced the risk of HE (Iebba et al., 2018).

Identifying specific gut microbiota provides new strategies for clinical diagnosis, treatment, and eventually weighing the prognosis of HE. In this regard, in hospitalised patients with cirrhosis, dysbiosis on admission (mainly changes in Proteobacteria constituents) was associated with increased risk of extra-hepatic organ failure, acute liver failure, and death, independent of clinical factors (Bajaj et al., 2019b).

Stool and salivary unique microbiome patterns predicted readmission and mortality at 90 days in cirrhotic patients, respectively (Bajaj et al., 2015b).

Sung et al. profiled dynamic changes in gut microbiomes of cirrhotic patients with overt HE at the acute episode before treatment, 48–72 h after active treatment, and the inactive stage (2–3 months after the episode) by comparing them with healthy individuals and patients with compensated cirrhosis. During acute hepatic encephalopathy (AHE), gut microbiome diversity and relative abundance of *Bacteroidetes* phylum diminished, whereas *Firmicutes, Proteobacteria*, and *Actinobacteria* increased. Moreover, the relative abundance of three species (*Alistipes*, *Bacteroides*, *Phascolarctobacterium*) and five operational taxonomic units (OTUs) (Clostridium-XI, *Bacteroides*, *Bacteroides*, *Lactobacillus*, Clostridium sedis) found during AHE were respectively associated with HE recurrence and overall survival during the subsequent 1-year follow-up (Sung et al., 2019).

Finally, in a prospective study involving 49 cirrhotic patients, Bloom et al. found eight species significantly less abundant in those patients with a history of OHE (*Anaeromassilibacillus species*, *Anaerostipes caccae*, *Bacteroideseggerthii*, *Clostridium species*, *Faecalicatena contorta*, *Holdemaniafiliformis*, *Neglecta timonensis*, and *Ruminococcus species*). However, none of the species was able to predict the future event of OHE. Moreover, they found an inverse correlation between bacterial species producing SCFAs and cirrhosis severity and lower concentrations of six specifical fecal SCFAs (acetate, propionate, butyrate, isobutyrate, valerate, and succinate) in patients with a history of OHE, thus supporting a crucial role of these metabolites in HE pathogenesis (Bloom et al., 2021).

## Therapy

Given the fundamental role of gut microbiota alteration in HE development, it is not surprising that most therapeutic strategies recommended by current guidelines primarily target gut microbiota or their bioproducts.

### Lactulose

Lactulose, a synthetic non-absorbable disaccharide, is part of the therapeutic armamentarium to treat HE since its first trials in the 1960s (Elkington et al., 1969). Behind the cathartic effect that reduces the contact time between luminal contents and the intestinal mucosa, lactulose lowers colonic pH creating a hostile environment for urease-producing gut flora and stimulating growth-acid-resistant, non-urease producing species. Furthermore, it reduces the absorption of ammonia by non-ionic diffusion. In 2014, the European and American Associations for the Study of the Liver (EASL/AASLD) published a joint practice guideline in which they recommended lactulose as the treatment of choice for OHE and secondary prevention after an index event (Vilstrup et al., 2014).

Despite its effect on ammonia production and improvement of HE symptoms, evidence linking the impact of lactulose on species richness in the gut microbiota remains conflicting. In earlier studies using stool culture, lactulose administration altered the relative abundance of certain gut bacteria, especially acidophilic, urease-deficient bacteria, such as *Lactobacilli* and *Bifidobacteria* (Vince et al., 1974; Merli et al., 1992). More recent studies based on culture-independent methods failed to demonstrate significant differences in composition or diversity of gut microbiota associated with lactulose administration or withdrawal (Bajaj et al., 2012b). Interestingly, patients who responded to lactulose treatment had a favorable modification of bacterial taxa. A recent randomised controlled trial conducted in patients with HE found significant differences between lactulose responders and non-responders in *Actinobacteria*, *Bacteroidetes*, *Firmicutes*, and *Proteobacteria* (Wang et al., 2019).

The apparent disconnection between reduction in blood ammonia and microbial changes, found in some studies, could be related to microbial changes below the detectable threshold or the relatively low sample size of the studies.

### Antibiotics

Antibiotics are presumed to exert therapeutic effects by decreasing colonic populations of urease-producing bacteria and, in combination with lactulose, are historically a mainstay of HE treatment. Over time, the prescribing trends evolved from chlortetracycline in the 1950s to neomycin and others now, with antibiotics generally falling out of favor because of severe side effects.

Rifaximin is a common antibiotic with broad-spectrum activity against aerobic and anaerobic Gram-positive and Gram-negative bacteria. The administration of rifaximin in patients with HE improves hyperammonaemia, endotoxemia, cognitive dysfunction and stimulates the immune system (Takaya et al., 2018; Mangas-Losada et al., 2019). According to current clinical practice guidelines, rifaximin is recommended as add-on therapy to prevent OHE recurrence, although it is also indicated in combination with lactulose in patients with overt HE.

More recent studies demonstrated that rifaximin impacts the function or activities of the gut microbiota by increasing serum levels of long-chain fatty acids and carbohydrate metabolism intermediates in patients with minimal HE and favorably affect serum proinflammatory cytokine. Furthermore, rifaximin in patients with HE has been associated with reduced gut ammonia-production via the action of glutamine and changes in the metabolism of bacteria-produced agents, such as lipopolysaccharide and secondary bile acid (deoxycholic acid) that contribute to maintaining normal gut microbiota levels (Bajaj, 2016; DuPont, 2016; Kang et al., 2016).

Regarding the effects on the gut microbiota composition, rifaximin is associated with a modest decrease in *Veillonellaceae* and an increase in *Eubacteriaceae*. Furthermore, rifaximin diminished the diversity and abundance of ammonia-producing bacteria such as *Clostridium* and *Streptococcus*, a risk factor for HE (Bajaj et al., 2013; Zuo et al., 2017). Nevertheless, although the favorable modulation of the microbiome by rifaximin in patients with HE was effective, there was no significant change in the overall relative abundance of bacteria (Kawaguchi et al., 2019). A newer agent currently used in clinical trials for the treatment of HE is nitazoxanide, a broad-spectrum antibiotic and antiparasitic agent with activity against gut anaerobes. However, studies on the effect of microbiota composition are still lacking (Glal et al., 2021).

### Probiotics

The World Health Organization defines probiotics as “live microorganisms that confer a health benefit on the host” (Hotel and Cordoba, 2001). Probiotics, with their pleiotropic effects, may be helpful to treat HE for their ability to suppress bacterial urease activity, lower ammonia absorption through pH reduction, modulate the immune response, and reduce intestinal permeability and uptake other toxins (indoles, oxindoles, phenols, and mercaptans). Furthermore, probiotics enhance the hepatic clearance of ammonia and other toxins by lowering gut-derived inflammatory signalling and oxidative stress in the liver (Solga, 2003). The most utilised probiotics include strains of lactic acid-producing bacilli (i.e., *Lactobacillus* and *Bifidobacterium*), non-pathogenic strains of *E. coli* (i.e., *E. coli Nissle* 1917), *Streptococcus salivarius*, a non-pathogenic strain of yeast (i.e., *Saccharomyces boulardii*), and a mixture of strains like VSL#3, which consists of eight different probiotic strains*: Streptococcus salivarius subp. thermophilus*, *Bifidobacterium breve*, *B. longum*, *B. infantis*, *Lactobacillus acidophilus*, *L. plantarum*, *L. paracasei*, and *L. bulgaricus*.

Several studies addressed the effect of probiotics, alone or in combination with standard therapy, in treating both MHE and OHE, with conflicting results. In MHE patients receiving probiotic Lactobacillus GG (LGG), Bajaj et al. described a significant improvement of dysbiosis characterised by the reduction of relative abundance of *Enterobacteriaceae* and increase in beneficial autochthonous taxa of *Lachnospiraceae* and Clostridiales Incertae Sedis XIV, (Bajaj et al., 2014a).

Treatment based on probiotics containing *C. butyricum* and *B. infantis* enriched Clostridium cluster I and *Bifidobacterium* abundance and decreased *Enterococci* and *Enterobacteriaceae* in MHE patients with HBV cirrhosis. Additionally, probiotic treatment was also associated with reducing venous ammonia and improved cognition (Xia et al., 2018).

The mixture of probiotics VSL#3 was found non-inferior to the standard therapy with lactulose in improving MHE and effectively preventing HE in patients with cirrhosis. (Lunia et al., 2014; Pratap Mouli et al., 2015). Moreover, a daily intake over 6 months significantly reduced the risk of hospitalisation for OHE. (Dhiman et al., 2014). On the other hand, Marlicz et al. did not find differences in incidence and grade of HE, assessed with critical flicker frequency, during probiotic supplementation (Marlicz et al., 2016). In the past years, multiple clinical trials and case reports have demonstrated the efficacy of symbiotics in HE treatment. The association of probiotics and fermentable fiber significantly increased the faecal content of non-urease-producing *Lactobacillus* species and was associated with a reduction in blood ammonia levels and reversal of MHE in 50% of patients (Liu et al., 2004). Furthermore, a 60 days treatment with a combination of *Bifidobacterium* and fructooligosaccharides was associated with a significant improvement of psychometric tests and blood ammonia levels when compared with the lactulose group in patients with HE (Malaguarnera et al., 2010).

Unfortunately, data from clinical trials on the use of probiotics to treat HE are difficult to compare because of differences in probiotic strains and delivery methods used, heterogeneity in study design and addressed endpoints (improved quality of life, progression from MHE to OHE, ammonia and endotoxemia reduction, gut microbiota modulation) (Khoruts et al., 2020). Finally, the relatively small number of colony-forming units in most commercial probiotic formulations hamper the optimistic conclusion that probiotics are sufficient to overtake the resident microbial community structure of cirrhosis and HE (Woodhouse et al., 2018).

A systematic review of 21 intervention trials including 1,420 participants indicated that probiotic supplementation vs placebo or no treatment reduced HE adverse events (including OHE development) and improved quality of life by lowering plasma ammonia concentration (Dalal et al., 2017). A more recent meta-analysis, including 14 randomised controlled trials and 1,132 patients, concluded that probiotic treatment effectively decreases serum ammonia and endotoxin levels, improves MHE, and prevents overt HE development in patients with liver cirrhosis. In addition, probiotics are as helpful as lactulose for MHE patients (Cao et al., 2018).

More recently, a meta-analysis including 25 trials and 1,563 participants found that probiotics effectively reversed minimal HE and prevented episodes of overt HE compared with placebo or no treatment; however, the evidence was low to moderate quality (Dhiman et al., 2020). Thus, drawing a definite conclusion on the efficacy of probiotics in HE still represents a tricky challenge.

### Fecal Microbiota Transplantation

Fecal microbiota transplantation (FMT) refers to the transfer of stool from “healthy” donors to patients with disordered gut microbes, with the purpose to restore eubiosis (Vindigni and Surawicz, 2017).

In patients with HE, FMT may reduce ammonia synthesis by shifting the gut microbiota composition to bacterial taxa low in urease, diminishing ammonia uptake by re-establishing intestinal barrier integrity, and increasing ammonia clearance by improving liver function. Earlier studies on animal models correlated FMT with lower ammonia production in the gut, reduced risk of encephalopathy, and protective effect against carbon tetrachloride-induced acute hepatic dysfunction (Shen et al., 2015; Wang et al., 2017). Interestingly, if the donor was a patient with HE, FMT results in neuroinflammation and microbial ecological disorders (Liu et al., 2020).

In a paradigmatic case report, Kao et al. first demonstrated that serial FMT in a patient with mild HE improved the cognitive function, assessed with Stroop test and inhibitory control test (Kao et al., 2016).

Later, in a randomised controlled trial, Bajaj et al. demonstrated that FMT from a rationally selected donor (i.e., high *Lachnospiraceae* or *Ruminococcaceae*) in 10 cirrhotic patients suffering from recurrent OHE reduced hospitalisations, improved cognition, and dysbiosis compared to standard of care (Bajaj et al., 2017). Similar results were obtained in a phase 1 trial using FMT via oral capsules in recurrent OHE. Post-FMT, cognitive performance improved, and duodenal mucosal diversity increased with higher *Ruminococcaceae*, *Bifidobacteriaceae*, and lower *Streptococcaceae* and *Veillonellaceae* (Bajaj et al., 2019a).

Recently, a systematic meta-analysis comprising two randomised clinical trials, three case reports, and three rodent studies highlighted the association between FMT and improved neurocognitive tests, lower hospital readmission rate, and a reduction in serious adverse events (Madsen et al., 2021). Despite the potential benefits, the risk of infections, likely due to lack of donor screening, burdens FMT and limits its use in the context of clinical trials.

## Conclusion

Moving from the pioneering era of culture-dependent studies, the connection between microbes, inflammation, and metabolic pathways in the pathogenesis of HE is becoming increasingly clear. PCR-based deep-sequencing technologies and metagenomic approaches are potent methods for studying microbiota and have provided high phylogenetic resolution of microbial communities inhabiting the gastrointestinal tract and their connection with the disease. However, they have substantial limitations. First, they unselectively detect microbes regardless of their viability, and different depths of sequencing lead to varying levels of selectivity. Furthermore, the results are based on the relative read abundances of microbial species in a given sample and thus do not provide exhaustive information on the function and dynamics of human-associated microbial ecosystems.

The recent evidence that viruses and fungi are active components of the gastrointestinal microbial ecosystem suggests that we are now starting to gain insight into the complexity of this organ. *In-vitro* models that resemble the human microbial environment, new methods to isolate and culture of previously unculturable bacteria, and emerging approaches to the study of the virome and mycome are now available. They will probably fill the gaps in our understanding of the microbiome’s role in maintaining health and developing diseases. The next challenge is to apply this understanding to develop new therapeutic strategies that target the microbial ecosystem based on the patient’s microbiome fingerprint.
